# Supplementary material for: Fix or freeze? Spectral differences arising from tissue preparation in chemical imaging
Source: Analyst. 2026 May 12;151(13):3721–32. doi: 10.1039/d6an00137h (PMC13177295; doi:10.1039/d6an00137h)
Supplement: AN-151-D6AN00137H-s001 [file AN-151-D6AN00137H-s001.pdf]

Supplementary Information (SI) for Analyst.
This journal is © The Royal Society of Chemistry 2026

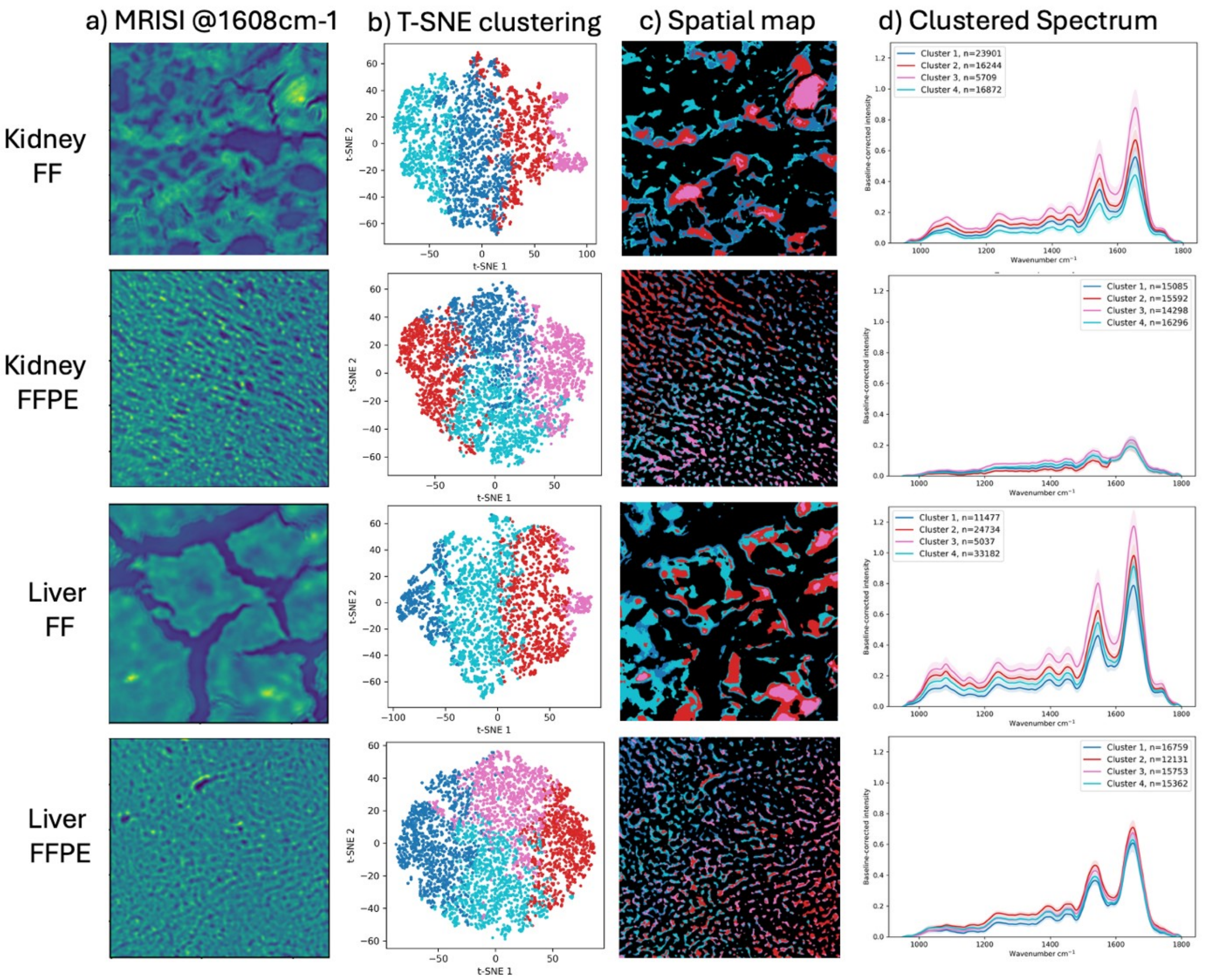

Figure S1. Pixel-resolved spatial clustering and spectral characterization of MIRSI data in FF and FFPE tissues.

Representative mid-infrared spectrochemical imaging (MIRSI) results from rat kidney and liver tissues prepared using fresh-frozen (FF) and formalin-fixed paraffin-embedded (FFPE) protocols. For each sample type (Kidney FF, Kidney FFPE, Liver FF, and Liver FFPE), four panels are shown: a) MIRSI image at 1608 cm^{-1} (Amide I region), b) t-SNE embedding of pixel-resolved spectra following PCA-based dimensionality reduction, c) corresponding spatial cluster map, and d) cluster-averaged spectra (mean ± standard deviation).

MIRSI images demonstrate the capability of capturing spatially resolved biochemical information at the pixel level. While t-SNE reveals spatial heterogeneity across tissue regions, cluster-averaged spectra show largely consistent spectral patterns within a region of interest (ROI) with variations primarily in band intensity. This indicates that the observed heterogeneity within an ROI is dominated by intensity differences rather than spectral profile changes, supporting that it is the chemical composition but not the thickness effects that drive the preparation-dependent spectral pattern differences.
